# Retromer-Mediated Trafficking of Transmembrane Receptors and Transporters

**DOI:** 10.3390/membranes5030288

**Published:** 2015-07-06

**Authors:** Stine C. Klinger, Piotr Siupka, Morten S. Nielsen

**Affiliations:** The Lundbeck Foundation Initiative on Brain Barriers and Drug Delivery, Department of Biomedicine, Aarhus University, 8000 Aarhus C, Denmark; E-Mails: siupka@biomed.au.dk (P.S.); mnbiomed.au.dk (M.S.N.)

**Keywords:** retrograde, trafficking, transmembrane, receptor, transporter, retromer, TGN, endosomes, sortilins, MPRs

## Abstract

Transport between the endoplasmatic reticulum, the Golgi-network, the endo-lysosomal system and the cell surface can be categorized as anterograde or retrograde, describing traffic that goes forward or backward, respectively. Traffic going from the plasma membrane to endosomes and lysosomes or the trans-Golgi network (TGN) constitutes the major retrograde transport routes. Several transmembrane proteins undergo retrograde transport as part of a recycling mechanism that contributes to reutilization and maintenance of a steady-state protein localization. In addition, some receptors are hijacked by exotoxins and used for entry and intracellular transport. The physiological relevance of retrograde transport cannot be overstated. Retrograde trafficking of the amyloid precursor protein determines the distribution between organelles, and hence the possibility of cleavage by γ-secretase. Right balancing of the pathways is critical for protection against Alzheimer’s disease. During embryonic development, retrograde transport of Wntless to the TGN is essential for the following release of Wnt from the plasma membrane. Furthermore, overexpression of Wntless has been linked to oncogenesis. Here, we review relevant aspects of the retrograde trafficking of mammalian transmembrane receptors and transporters, with focus on the retromer-mediated transport between endosomes and the TGN.

## 1. Introduction

Secretion from the endoplasmatic reticulum (ER) sends proteins towards the Golgi complex, the trans-Golgi network (TGN), the plasma membrane or the endo-lysosomal system, and is known as anterograde traffic. Transport in the opposite direction is called retrograde, but is often not defined beyond that. Transmembrane proteins, such as receptors, transporters, proteases and soluble NSF attachment protein receptors (SNAREs) are constantly being exported to and anchored at the plasma membrane [1,2,3,4]. The balancing of outgoing and incoming traffic is essential for maintaining the functions of both the endosomal system and the TGN. Retrograde traffic includes the transport going from the endosomal system toward the TGN or from the Golgi to the ER. Here, we focus on the retromer-mediated retrograde transport going from endosomes towards the TGN, but we also briefly mention the retromer-mediated transcytosis taking place in polarized epithelial cells.

**Figure 1:**
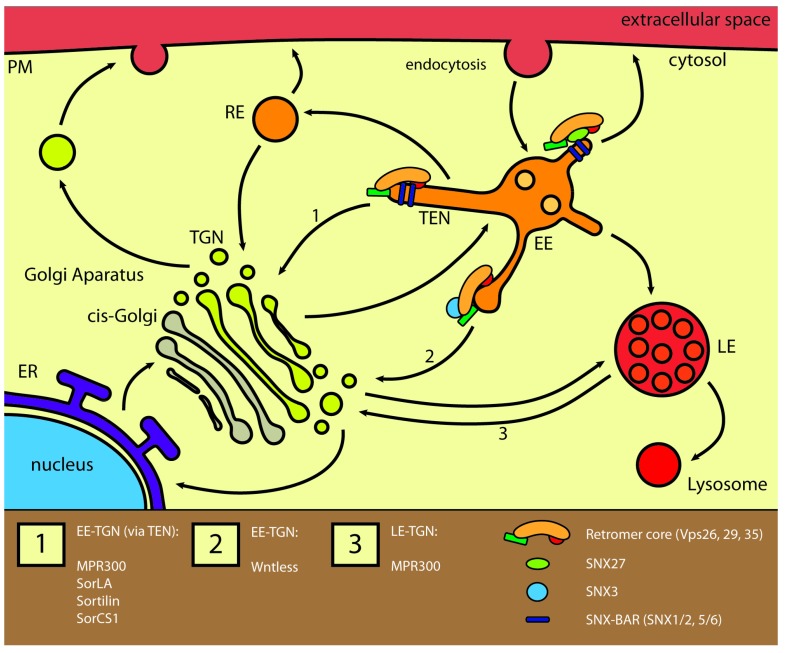
Retrograde trafficking of receptors. Retrograde trafficking can be divided into routes going from early endosomes towards the trans-Golgi network (TGN), from late endosomes or via recycling endosomes. The most common route goes from early endosomes and is mediated by the SNX-BAR-retromer as well as the SNX3-retromer. The SNX-BAR-retromer mediates trafficking of MPR300 and the sortilin family members via the tubular-endosomal network (route 1), while the SNX3-retromer mediates trafficking of Wntless (route 2). The route from late endosomes is mainly used by MPR300 independent of retromer (route 3). Abbreviations: PM: plasma membrane; ER: endoplasmatic reticulum; RE: recycling endosome; EE: early endosome; LE: late endosome; TEN: tubular-endosomal network.

After internalization, proteins are transported to early endosomes, which make up the first step along the retrograde pathway, and is an essential way station before any following step [5] (Figure 1). From early endosomes, transport continues to late endosomes and eventually lysosomes for degradation, to the plasma membrane, or to the TGN. Transport to the plasma membrane is divided into the fast, direct pathway and the slower pathway, going via recycling endosomes [6,7]. Likewise, transport to the TGN may go through recycling endosomes or even late endosomes, and some proteins continue towards the ER (Figure 1) [1,2,8]. Neuronal retrograde traffic is simply defined as traffic going towards the cell body, regardless of the compartments involved [9]. Given the size of neurons, their retrograde traffic is highly important for maintaining necessary gradients of proteins and signaling molecules. This topic has been excellently covered in other reviews [9,10,11] and will not be further discussed here.

A well-functioning retrograde machinery is essential in order to maintain the correct distribution of lipids and proteins in the TGN as well as endosomes (reviewed in [1]). Defects are associated with a range of disorders, including Alzheimer’s and Parkinson’s diseases [12,13]. Likewise, secretion of Wnt proteins depends on recycling of the Wntless sorting receptors and is essential for tissue patterning during development [14]. Several plant, bacterial and viral proteins also rely on endocytosis and retrograde transport for cellular entry and following correct delivery to target compartments. This includes among others *Shigella dysenteriae* Shiga toxin, *Vibrio cholera* Cholera toxin and HIV-1 envelope protein [15,16,17]. Studies of the cellular uptake of the *Ricinus communis* toxin ricin gave the first description of the retrograde pathway in 1975 [18], and several proteins have since been found to utilize this pathway. The early endosome-to-TGN pathway is the choice for most receptors and transporters, while the deliverers of lysosomal hydrolases, the mannose-6-phosphate receptors, are the only known example of transmembrane receptors to traffic from late endosomes (Figure 1).

## 2. The Retromer Complex and Trafficking

### 2.1. Retrograde Transport from Endosomes towards the TGN

The transport from endosomes towards the TGN is generally considered to consist of several parallel pathways, going from early, late or recycling endosomes. The clear division of endosomes into specific compartments is somewhat misleading, as several studies have concluded that endosomes mature gradually through processes of fusion and fission, and therefore cannot be considered discrete compartments [19,20,21]. The terms early and late endosomes are therefore solely used for explanatory purposes in this text. The highly dynamic endosomal system may be an explanation of the convergent functions of the many accessory proteins involved in the retrograde transport of mammalian receptors and transporters.

**Figure 2:**
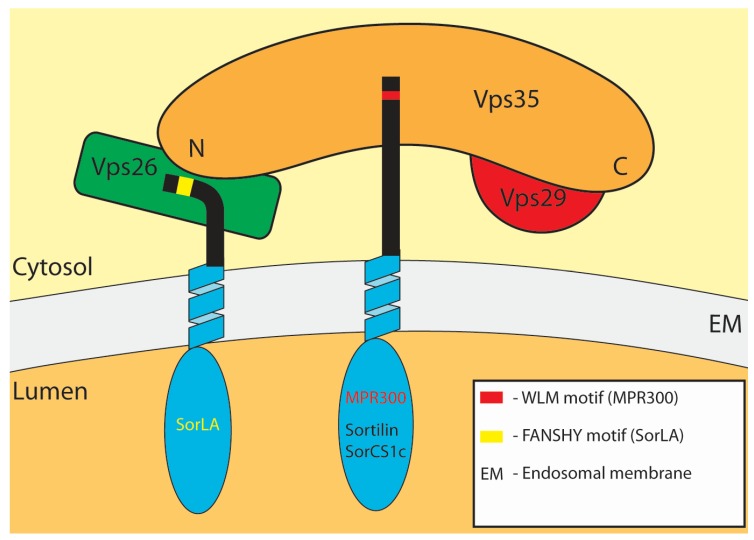
Retromer interactions with type-1 receptors. The retromer core complex consists of Vps35, Vps26 and Vps29. The banana-shaped Vps35 interacts with Vps26 through its N-terminal and Vps29 through the C-terminal. Located at endosomes, type-1 receptors have been reported to bind to Vps35 as well as Vps26. MPR300, sortilin and SorCS-1C bind Vps35; MPR300 via a WLM motif while the binding motifs in sortilin and SorCS-1C are unknown. Sortilin may also interact with Vps26, whereas SorLA has only been reported to bind Vps26 through a FANSHY motif.

Generally, adaptors recognize their cargo, and mediate transport, assisted by a broad range of accessory proteins involved in scission from the donor compartment, transport along the cytoskeleton and fusion with the target compartment. The retromer is a central player in the process of retrograde trafficking. It was initially discovered in yeast, where it mediates retrieval of the vacuolar protein sorting (Vps) 10 protein (Vps10p), which transports acidic hydrolases, and in absence of retromer will be delivered to the vacuole [22,23]. It consists of five subunits; Vps26p, Vps29p, Vps35p, Vps5p and Vps17p. Vps26p, Vps29p and Vps35p form the highly conserved cargo-selecting subcomplex while the latter two mediates membrane interaction [22,24]. The mammalian counterpart likewise consists of two subcomplexes; a Vps26-Vps29-Vps35-trimer and a membrane-interacting dimer. The dimer consists of the Vps5p orthologues sorting nexin (SNX) 1 or SNX2 and the Vps17p orthologues SNX5 or SNX6 [25,26,27]. Vps35 is the largest member of the cargo-binding subcomplex. The C-shaped subunit interacts with the smallest subunit Vps29 through the C-terminal region and with Vps26 at the N-terminal end [28] (Figure 2). Vps35 is generally known as the main cargo-binding subunit, although interactions between sortilins and Vps26 have been reported [29,30]. Vps26 exists as three paralogues, Vps26A, Vps26B and Down’s syndrome critical region (DSCR3), of which the first two are highly similar and best characterized [31,32]. Vps29 is essential for interaction of the Vps26-Vps35-Vps29 subcomplex with the SNX dimer and functions as a scaffold for retromer assembly by binding the C-terminal half of Vps35 [33]. The trimer does not interact directly with membrane lipids, but remains associated with endosomes due to interactions with the small GTPase Rab7 [33,34]. The membrane-interacting subcomplex can be formed from any combinations of SNX1 or SNX2 and SNX5 or SNX6 [26,27] (or SNX32, according to a recent review [31]). All these SNXs contain two membrane-binding domains; a BAR (Bis/amphiphysin/Rvs) domain, which senses membrane curvature, and a phosphoinositide-binding PX (phox homology) domain, and therefore belong to the SNX-BAR family [35,36]. The main target of PX-domains is phosphatidylinositol 3-phosphate (PI3P), which is highly enriched in early endosomes [37]. The C-terminal BAR-domains form a C-shaped structure upon dimerization. The inner part interacts electrostatically with the membrane and senses the curvature, but the BAR-domains can also induce and stabilize curvature and as such participate in forming the tubular-endosomal network (TEN) [38,39]. The retromer is found on early and late endosomes due to interactions with the GTPases Rab5 and Rab7, respectively [34]. Rab7 has been proposed to bind directly to Vps35, an interaction enhanced dramatically by the subsequent binding of Vps26 [40].

In addition to the above-described retromer, which is the complex mainly referred to (and also known as the SNX-BAR-retromer), two additional retromer complexes exist, based on the core complex Vps26-Vps29-Vps35 in combination with either SNX27 or SNX3 [41,42,43]. The SNX27-retromer has been shown to mediate fast recycling of the β2-adrenergic receptor and the copper transporter Menkes protein from endosomes directly to the plasma membrane without crossing the TGN [43,44,45]. The SNX3-retromer mediates endosome-to-TGN transport of the Wntless receptor [41,46], but has also been suggested to participate in recycling of the transferrin receptor [47].

Retromer-mediated trafficking is assisted by a broad range of accessory proteins, enabling vital events such as recruitment, membrane scission and fusion, docking and transport along the cytoskeleton. Some factors are essential, while others are only required for certain cargos (reviewed in [3,4,8,31,48,49]).

Clathrin forms coated pits that, in addition to the plasma membrane, have been found on early as well as more mature endosomes, and are necessary for the retrograde trafficking originating there [50,51,52]. The transport from late endosomes to the TGN is unaffected by clathrin inactivation [53]. The whole sequence of events leading to the formation of the cargo-loaded tubules at the early endosome is far from understood. Studies by McGough and Cullen [54] have shown that the formation of retrograde carriers from early endosomes begins with clathrin binding and organizing cargo on a flat lattice. The DNAJ protein RME-8 is then thought to bind SNX1 and thereby recruit the retromer complex [54]. The SNX BAR-domains initiate membrane folding and generate the tubular structures of the TEN. At the same time, SNX1 attracts the clathrin disassembly factor Hsc70, and thus couples clathrin coat shedding with retromer assembly [55,56]. In addition to SNX1, RME-8 also binds the FAM21 subunit of the Wiscott-Aldrich syndrome protein and SCAR homologue (WASH) complex. The WASH complex recruits Arp2/3 to generate patches of actin polymerization, producing a pushing force that leads to elongation of endosomal tubules [57]. The interactions with RME-8 maintain the cargo-binding Vps-trimer, the curvature-generating SNX-dimer and the tubule-forming WASH complex close [58].

An additional force, enabling tubule elongation and eventually scission, comes from SNX5 or 6 interacting with the p150^Glued^ subunit of the dynein-dynactin complex, enabling the endosome-associated retromer to “walk” along the microtubule [59,60,61]. Arriving at the TGN, tethering factors link the transport vesicle to the acceptor compartment where after SNARE complexes mediate the actual fusion of the two membranes (reviewed in [4,8,49]).

Retromer-dependent retrieval from maturing endosomes takes place independent of clathrin [54] while retromer has not been detected at mature late endosomes. Late endosomal sorting by the GTPase Rab9 represents an alternative to the retromer [62], and these somewhat overlapping functions may reflect the fatality of well-functioning proteins being sent to lysosomes.

### 2.2. Transcytosis

The process of transcytosis, as seen in epithelial and endothelial cells, is essential for maintaining polarity of the cells. In addition, transport of macromolecules and micronutrients, including IgA and vitamin B12, across barriers, provides a mechanism for crossing an otherwise impermeable cellular barrier [63,64,65]. Capillaries are a well-known site for transcytosis (reviewed in [64]), and transcytosis across the blood-brain barrier could prove to be a potential method for delivery of therapeutic drugs. In addition to its role in retrograde trafficking, the retromer is also involved in basolateral-to-apical transcytosis of the polymeric immunoglobulin receptor in Madin-Darby canine kidney (MDCK) cells [65]. In fact, recent results indicate that in MDCK cells, and perhaps also other epithelial or endothelial cell lines, the main function of the retromer is transcytosis rather than retrograde transport [66].

## 3. Retromer-Binding Receptors and Transporters

### 3.1. Cation-Independent Mannose-6-Phosphate Receptor (MPR300)

The mammalian cation-independent mannose-6-phosphate receptor (CI-MPR or MPR300), is one of the most studied receptors using the retrograde retrieval pathway. The type-1 receptor is well-recognized for its role in delivery of soluble lysosomal hydrolases [67], and is characterized by the presence of 15 mannose-6-phosphate homology repeats in the extracellular domain, of which four are involved in mannose-6-phosphate-tagged ligand-binding [68,69,70,71,72,73]. MPR300 binds mannose-6-phosphate-tagged acidic hydrolases in the TGN and transports them to endosomes, where they are released in a pH-dependent manner [74]. The transport is mediated by clathrin, the Golgi-localized, γ-ear containing, Arf-binding family of proteins (GGAs), which recognizes a DXXLL motif in the cytoplasmic tail, and AP-1, which can interact with several binding sites including the tyrosine-based YXXØ (where Ø is a bulky hydrophobic amino acid) and D/EXXXLL/I [74,75,76,77]. The GGAs and AP-1 appear to cooperate, as depletion of AP-1 leads to a loss of MPR300 and GGA2 in clathrin coated transport intermediates [78]. Delivery of lysosomal enzymes is essential for the maintenance of proper functioning lysosomes, and defective mannose-6-phosphate tagging causes the lysosomal storage disorder I-cell disease (reviewed in [79]). After successful delivery of lysosomal hydrolases, the receptors are returned to the TGN for another round of ligand binding. The retrograde transport goes from early as well as late endosomes, and is mediated partly by the retromer complex, partly by Rab9 [80,81]. Rab9 mediates retrieval from late endosomes, while the retromer mainly sorts MPR300 from early or maturing endosomes [80,81,82,83,84]. TIP47 was previously believed to assist Rab9 in the retrograde trafficking from late endosomes [85], but has since been shown not to colocalize with or affect the trafficking of MPR300 [86]. In addition, AP-1 and PACS-1 may also participate in the retrograde trafficking of MPR300 [87,88]. Several retrieval motifs are found in the cytosolic tail, reflecting the variety of adaptors and emphasizing the importance of receptor recycling [85,89,90]. Specifically, a WLM motif is responsible for interaction between MPR300 and the retromer Vps35 subunit [80,90] (Figure 2 and Table 1). The MPR trafficking pathways have a large similarity with the pathways for sortilin and Wnt trafficking, as described below.

**Table 1:** Receptors and transporters engaging in retromer-mediated traffic.

Receptors	Type	Ligands	Retromer sorting	Interacting retromer subunit	Receptor binding motif	References
CI-MPR (MPR300)	Type-I	M6P containing ligands: acidic hydrolases, TGF-β1, proliferin, granzyme B, thyroglobulin; Non-M6P containing ligands: IGF-II, retinoic acid, uPAR, plasminogen, serglycin	EE to TGN	Vps35	WLM	26
SorLA (LR11)	Type-I	LpL, apoE, apoA-V-DMPC, GDNF, GFRα1, uPA-PAI1 PAI-1, tPA-PAI-1, PDGF-BB, APP, SPAK, TrkB	EE to TGN	Vps26	FANSHY	30, 107
Sortilin	Type-I	LpL, pro-NGF, neurotensin, progranulin PGRN, IL6, apoE, PCSK9, delta-like homologue receptor, IL6, IFN-gamma, CNTF, apoAV, apoB100, APP	EE to TGN	Vps35	*Not known*	29,105
SorCS-1	Type-I	PDGF-BB, APP, sortilin	*Not known*	Vps35	*Not known*	108,109
pIgR	Type-I	IgA	Transcytosis	Vps35	*Not known*	65
Wntless (GPR177)	7TM	Wtn family proteins	EE to TGN	Vps35	FLM	120, 124
**Transporters**				**		
Menkes protein (ATP7A)	ATPase (8TM)	Cu^++^	Recycling to cell surface	Vps26	*Not known*	45, 132
Glut4	12 TM	Glucose	EE to TGN	*Not known*	*Not known*	128, 130
DMT1-II	12 TM	Divalent cation transporter	Recycling to cell surface	*Not known*	YLL	135, 136

### 3.2. Sortilins

The Sortilins are a group of 5 mammalian type-1 receptors named sortilin, SorLA and SorCS-1 to 3. They are characterized by having a luminal Vps10p-domain and a small cytoplasmic domain containing several known sorting motifs [91,92,93,94]. The receptors are involved in signaling, endocytosis and subcellular trafficking and have several physiological roles [95,96,97]. For instance, SorCS-1 has been associated with diabetes, SorLA is related to Alzheimer’s disease and lipoprotein trafficking, and sortilin is a cardiovascular risk gene and involved in amyloid precursor protein processing and neurotrophic factor signaling [95,98,99,100,101,102,103,104]. SorLA and sortilin have a particular high degree of similarity to MPR300 in their cytoplasmic domain. Like MPR300, sortilin and SorLA are involved in endocytic processes and TGN-to-endosome shuttling mediated by adaptor proteins. For sortilin, a tyrosine-based YXXØ-type motif is responsible for endocytosis, whereas a dileucine-like VL motif is essential for the TGN to endosome sorting [105]. The dileucine-like signal is part of tyrosine-based motif and it is involved in binding to µ1A in AP-1, but sortilin also binds to σ1B subunit in adipocytes by an extended DXXD-X12-DSXXXL motif [106]. SorLA is endocytosed in an AP-2 dependent process and is also involved in TGN-endosome shuttling. Unlike sortilin, both these sorting events seem to be controlled by a cluster of acidic amino acids [107]. Both sortilin and SorLA are subject to retrograde transport from endosomes to TGN by the retromer. The retrograde transport of sortilin takes place from early endosomes in a SNX1 dependent manner. Sortilin does not exit early endosomes via the TEN, but from short non-branched tubules without clathrin coats [83]. A later study has demonstrated that sortilin interacts with the retromer via Vps35 and perhaps also Vps26B [29]. The motif mediating this interaction in sortilin remains unknown (Figure 2 and Table 1). Like sortilin, down-regulation of SNX1 with siRNA, compromises the retrograde transport of SorLA to TGN and increases lysosomal degradation of the receptor [107]. This study does not describe the subcellular structure of the endosomal system wherefrom SorLA exits, but it might be similar to sortilin, as SorLA also interacts with Vps26. This binding is facilitated by the FANSHY sorting motif in SorLA [30]. SorCS-1 has several different cytoplasmic tails due to alternative splicing, and one of these variants, SorCS-1C, has been reported to bind Vps35 [108]. The study does, however, not report any retromer-mediated transport of SorCS-1, and accordingly, an earlier report demonstrate that none of the SorCS-1 splice variants seem to be involved in endosome-to-TGN shuttling [108,109].

### 3.3. The Wntless Receptor

The Wntless receptor, also known as GPR177 in mammals, is a large membrane-spanning receptor involved in the secretion of Wnt family proteins (Wnts) [14,110]. Wnts are small, highly conserved glycoproteins with central functions during development and homeostasis, including regulation of gene expression [14,111,112]. The Wnt receptor itself has, based on structural predictions, been suggested to have 7 or 8 transmembrane segments. The intracellular localization of both N- and C-terminus indicate an even number of membrane spanning domains [113].

The Wnt-binding domain appears to be a lipocalin-like domain found in the luminal loop right before the second membrane spanning helix [14,114]. Ligand binding may involve hydrophobic interactions, and depends on palmitoylation and glycosylation of residues within the ligands, as well as pH [112,115,116,117,118]. Wnts are bound by the Wntless receptor as early as in the ER, where post-translational modification is still taking place. Consequently, Wntless receptors are found throughout the Wnt secretory pathway, from the ER over Golgi apparatus to secretory vesicles and the plasma membrane [14,114,115,119]. After delivery of Wnts at the plasma membrane, the receptors are internalized for another round of ligand binding and secretion [120,121,122,123]. The clathrin-dependent endocytosis is mediated by Rab5 and AP-2, which binds the Wntless receptor via an YXXØ-type motif in one of the intracellular loops [41,122,124,125]. During endosomal passage, Rab5 and AP-2 are replaced by the retromer complex [120,121,123]. Vps35 binds the Wntless receptor, most likely through the tripeptide FLM in the third intracellular loop [14,90] (Table 1). In contrast to other receptors mentioned in this review, retrograde trafficking of the Wntless receptor is not mediated by the SNX-BAR-retromer, but by the SNX-3-retromer complex [41,46]. The complex exits from early endosomes in small vesicles independent of the TEN created by SNXs with BAR domains [14,41,46]. After delivery to the TGN, the Wntless receptor is further recycled to the ER in COPI-coated vesicles [126].

### 3.4. Other Receptors and Transporters

Several transmembrane transporters follow the retrograde transport route from endosomes to the TGN, although less detail about the mechanism is known (Table 1). So far, no one has demonstrated direct retromer-mediated endosome-to-TGN trafficking of any transporter. One such transporter is Glut4, an insulin-regulated glucose transporter that is important for glucose transport in adipocytes and muscle cells [127,128]. After internalization, some Glut4 has been shown to return to TGN subdomains enriched in Syntaxin 6 and 16 [129]. This, combined with data demonstrating that Vps26 is essential in order to rescue Glut4 from lysosomal degradation, makes it likely that the retromer is involved in retrieval of Glut4 [130]. The report also demonstrated that insulin induced dissociation of retromer components from the low-density microsomal membranes of adipocytes and thereby regulated the fate of Glut4. The human copper transporter, ATP7A (also known as Menkes protein) is another example of a retrograde transported transporter [131,132,133]. ATP7A is recycled between TGN and endosomes, but this anterograde and retrograde transport seems to depend on interaction with AP-1 and AP-2 [134]. Nonetheless, the Vps26-Vps29-Vps35 retromer core complex is also involved in trafficking of ATP7A in combination with SNX27 and the WASH complex. This interaction seems to influence the transport of ATP7A from endosomes to the cell surface, and hence prevent lysosomal degradation [45]. Likewise, a non-epithelial isoform of the divalent metal transporter, DTM-II, has been shown to depend on the retromer for recycling back to the cell surface [134,135,136]. The trafficking involves the tyrosine-based YLL signal, and it is uncertain if the retromer also facilitates retrograde transport to the TGN [135]. Finally, the retromer is involved in the transcytosis of pIgA receptors in MDCK cells [65,137]. Though a small fraction of the retromer co-localize with MPR300 in MDCK cells, the majority is co-localized with the pIgA receptor, which is associated with a specialized early endosome-derived transcytosis pathway. This indicates that the primary function of the retromer in epithelial cells is receptor transcytosis rather than early endosome-to-TGN retrograde transport [66]. 

## 4. Concluding Remarks

Since its discovery, the retromer has emerged as a trafficking complex participating in the endosome-to-TGN trafficking of many receptors, transporters and other types of proteins. Recently, several studies have confirmed that the retromer core complex can mediate transport between other compartments as well, a diversity induced by interactions with different SNXs. Likewise, subunits other than Vps35 have been shown to bind cargo, and this also seems to affect the transport, in particular the budding process, giving rise to morphologically distinct tubules or carriers. More details about the interactions between retromer subunits and their cargo will undoubtedly enhance our understanding of the biology of the retromer complex. This includes a greater appreciation of the roles of the individual subunits and the motifs they bind, as well as the mechanisms behind the different budding processes. Without exemption, all receptors and adaptors sorted by the retromer complex play important roles in development of various diseases, and correct trafficking and recycling is crucial for their function. A better comprehension of their interactions with the retromer will therefore greatly improve our chances of understanding the molecular mechanisms underlying certain pathogenesis.
